# Improved Eating Behaviour and Nutrient Intake in Noncompliant Patients with Phenylketonuria after Reintroducing a Protein Substitute: Observations from a Multicentre Study

**DOI:** 10.3390/nu11092035

**Published:** 2019-08-30

**Authors:** Benjamin Green, Yusof Rahman, Sarah Firman, Sarah Adam, Fiona Jenkinson, Claire Nicol, Sandra Adams, Charlotte Dawson, Louise Robertson, Carolyn Dunlop, Alison Cozens, Gary Hubbard, Rebecca Stratton

**Affiliations:** 1 Medical Affairs, Nutricia Advanced Medical Nutrition, Wiltshire BA14 0XQ, UK; 2 Guy’s and St Thomas’ Hospital, London SE1 9RT, UK; 3 Royal Hospital for Children, Glasgow G51 4TF, UK; 4 Royal Victoria Infirmary, Newcastle NE1 4LP, UK; 5 Queen Elizabeth Hospital, Birmingham B15 2TH, UK; 6 Royal Hospital for Sick Children, Edinburgh EH9 1LF, UK; 7 Faculty of Medicine, University of Southampton, Southampton SO14 0DA, UK

**Keywords:** phenylketonuria, PKU, compliance, noncompliance, micronutrient, nutrient intake, eating behaviour, nutritional status, mood

## Abstract

Noncompliance is widespread in adults with PKU and is associated with adverse metabolic, nutritional and cognitive abnormalities. Returning to the PKU diet is important for this at-risk population, yet for many this is challenging to achieve. Strategies that ease the return to the PKU diet, while offering nutritional and cognitive advantages, are needed. Twelve PKU adults (33.7 ± 2.6 years), who had been noncompliant for 4.5 years (range: 1 to 11 years), took 33 g of a low-volume, nutrient-enriched, protein substitute daily for 28 days. Outcomes of eating behaviour, nutrient intake and mood were assessed at entry (baseline, days 1–3) and after the intervention period (days 29–31). At baseline, intakes of natural protein and estimated phenylalanine were high (66.4 g and 3318.5 mg, respectively) and intakes of calcium, magnesium, iron, zinc, iodine and vitamin D were below country-specific recommendations. With use of the experimental protein substitute, natural protein and estimated phenylalanine intake declined (*p* = 0.043 for both). Fat and saturated fat intakes also decreased (*p* = 0.019 and *p* = 0.041, respectively), while energy and carbohydrate intake remained unchanged. Micronutrient intake increased (*p* ≤ 0.05 for all aforementioned) to levels well within reference nutrient intake recommendations. Blood vitamin B_12_ and vitamin D increased by 19.8% and 10.4%, respectively. Reductions in anxiety and confusion were also observed during the course of the study yet should be handled as preliminary data. This study demonstrates that reintroducing a low-volume, nutrient-enriched protein substitute delivers favourable nutritional and possible mood benefits in noncompliant PKU patients, yet longer-term studies are needed to further confirm this. This preliminary knowledge should be used in the design of new strategies to better facilitate patients’ return to the PKU diet, with the approach described here as a foundation.

## 1. Introduction

Phenylketonuria (PKU) is a rare autosomal recessive disorder of amino acid metabolism [1]. Characterised by mutations in the hepatic enzyme phenylalanine hydroxylase (PAH), PKU is one of the most prevalent inborn metabolic disorders, with reported incidence rates around 1 in every 10,000 births [1]. Mutations of the PAH gene subsequently inhibit phenylalanine hydroxylation, a process pertinent for the conversion of phenylalanine (an essential amino acid) to tyrosine [2]. Consequently, blood, brain and body tissue concentrations of phenylalanine increase and accumulate. Without appropriate dietary management, the accumulation of phenylalanine concentrations can be toxic and manifest in a spectrum of neuroanatomical and neurophysiological alterations including cognitive impairment, behavioural disorders, microcephaly, physical and learning disability [3].

Management of phenylketonuria (PKU; OMIM 261600) is complex and predominantly dietary. In clinical practice, the objective of PKU dietary management is two-fold: foremost, to maintain concentrations of circulating phenylalanine at a level necessary to allow almost normal clinical outcome, while secondly providing a nutritionally complete diet [4]. The provision of protein substitutes (low or phenylalanine-free) acts as an integral adjunct to the dietary management of PKU to ensure this [5]. Composed primarily of L-amino acids, protein substitutes (when fully compliant) represent patients’ main source of non-phenylalanine nitrogen, supplying 70–85% of a patient’s daily protein requirements and often vitamins, minerals and docosahexaenoic acid (DHA) [6]. Without appropriate dietary management, phenylalanine accumulates and manifests in a myriad of complications including neurologic, neurocognitive and neuromotor impairment [1].

European guidelines recommend that phenylalanine restriction and protein substitute intake (herein referred to as the PKU diet) should be continued life-long [7], even though this is burdensome, considerably challenging and often difficult for patients to follow [8]. Because of this, many patients become noncompliant. In the context of PKU, noncompliant refers to deviation from the prescribed PKU diet and is commonly coupled with disengagement from metabolic follow-up [9]. Rates of noncompliance are highly prevalent in PKU, especially in older adolescents and adults [9]. Early reports suggest that 80% of UK and Australian adolescent and adult PKU patients have blood phenylalanine concentrations above recommended limits because of noncompliance [10]. This is reflected in the US where national data estimates 77% of adolescents and adults are noncompliant [11], with additional reports suggesting that 52% (of 625 PKU-related respondents) find it difficult to maintain the PKU diet [12]. More recently, 43% of adult patients in the UK admitted to not following the PKU diet [13]. Noncompliant patients are therefore typically not under strict dietary control, have a variable degree of natural protein restriction and tend to relax and/or completely discontinue taking protein substitutes [14,15].

Coupled with increased concentrations of circulating phenylalanine and thus poorer metabolic control, noncompliance may make patients at risk of nutritional deficiencies compared to fully compliant counterparts and healthy controls. Patients relaxing and/or stopping the PKU diet display insufficient nutrient intakes, despite compensatory increases in natural protein intake [15,16]. Insufficient intakes have been reported for vitamin B_12_, iron, zinc, vitamin D_3_, calcium, selenium, iodine [15,16] and long-chain polyunsaturated fatty acids (particularly DHA and arachidonic acid) [17,18]. Impairments in executive functioning, information processing (reaction times, attention) and mood (increased inhibition, anxiety, depression and low self-esteem) have also been reported in noncompliant PKU patients and likely transpire due to the pathophysiological consequences of disrupted phenylalanine hydroxylation [19,20]. Interestingly, reinstating a strict PKU diet may overturn these complications, while restoring metabolic control and nutritional adequacy. While simple in theory, research demonstrates the return to strict compliance (which includes 3–4 protein substitutes and severe natural protein restriction) is extremely difficult, with many patients again noncompliant after several months. In this sense, despite reports of improved quality of life, Bik-Multanowski et al. [21] reported that only 55% (29 of 53 patients) of adults were able to achieve strict dietary compliance for 3 months after returning to the PKU diet, and only 19% (10 patients) were able to follow the diet for 9 months [21].

Effort is needed to help build effective strategies with the potential to optimise the nutritional supply and cognitive stability of this at-risk population while improving metabolic control through greater compliance. Considering the difficulties observed when attempting to return to the PKU diet, and the nutritional and cognitive consequences of being noncompliant, a stepwise approach may prove beneficial with the overarching aim to move to full compliance over time. Reintroducing a low-volume protein substitute with elevated quantities of micronutrients that are characteristically low in a noncompliant population may be an advantageous starting point. Against this background, the present study explored this approach with outcomes of eating behaviour, nutrient intake and mood over 28 days in a population of noncompliant PKU adults.

## 2. Materials and Methods

### 2.1. Recruitment and Study Population

Patients with PKU were recruited across six specialist metabolic centres in the UK. Inclusion was restricted to patients over the age of 16 years, with blood phenylalanine levels of ≥600 µmol/L, taking a maximum of 1 protein substitute per day (equal to 20 g protein equivalent) and to have been noncompliant to dietary management for at least 1 month prior to data collection.

### 2.2. Study Design and Ethics

Patients attended their respective metabolic clinic on two occasions separated by 31 days, for review. Patients firstly observed a 3-day baseline period where usual eating behaviours, nutrient intake, selected nutritional biomarkers and mood were established. On day 4 of the study, patients began to take the low-volume, nutrient-enriched, protein substitute (herein the experimental protein substitute) daily for 28 days (intervention period). Composed of an adapted mixture of essential and non-essential amino-acids, the experimental protein substitute provided 20 g protein equivalent and elevated concentrations of micronutrients that are characteristically low in a noncompliant population. It was also enriched with DHA (100 mg per 33 g serving). The experimental protein substitute was presented in pre-packaged sachets (33 g) of citrus flavoured powder (PKU Synergy^©^, Nutricia Ltd, Liverpool, UK, L7 9PT.) to be consumed once daily for the 28-day intervention period. Details of the nutrition composition of the experimental protein substitute are provided in Table 1. Patients were recommended to reconstitute the powder in 100 mL of water and (where possible) to consume alongside natural protein and additional energy (e.g., with a meal) for optimal utilisation. During the intervention period, patients were instructed to maintain their usual feeding and physical activity practices. Eating behaviours, nutrient supply, selected nutritional biomarkers and mood were assessed again throughout days 29–31.

The National Health Service (NHS) Research Ethics Committee (Cambridge East—17/EE/0078) reviewed the experimental procedures and approved the study. The study was conducted in accordance with the Declaration of Helsinki of 1975, as revised in 2013, and ICH-Good Clinical Practice. All patients provided written informed consent before any study-related procedures were performed.

### 2.3. Eating Behaviour and Nutrient Intake

Eating behaviour and nutrient intake were evaluated over 3 consecutive days during baseline (days 1–3) and during the final 3 days of the intervention (endpoint, days 29–31) utilising detailed food records. Patients were requested to give full comprehensive recordings of all food, drink and protein substitutes consumed, weighing all items prior to and following consumption (if leftovers were present). Additional information deemed necessary included methods of preparation and cooking, names of branded products and condiment use. For homemade dishes, patients were asked to record individual ingredients and quantities for the whole dish, along with a brief description of cooking method and how much of the dish they consumed. One member of the research team examined all food records utilising the nutritional software package Nutritics (Nutritics Research Edition v5.042, Dublin, Ireland).

Food records were analysed for energy, macronutrient (carbohydrates, fats and protein [total protein, natural protein and protein from protein substitutes]), micronutrient, and phenylalanine intake. Phenylalanine intakes were estimated based on the gram of protein exchange system (1 g protein = 50 mg phenylalanine), a system commonly used in the PKU community [22]. Intakes of micronutrients were compared against the UK reference nutrient intakes (RNI) to determine nutritional adequacy [23].

### 2.4. Nutritional Biomarkers: Blood Phenylalanine, Tyrosine, Vitamin B12, Zinc and Vitamin D

Blood samples for phenylalanine (µmol/L), tyrosine (µmol/L), vitamin B_12_ (holotranscobalamin; pmol/L), zinc (µmol/L) and vitamin D (25-hydroxyvitamin D; nmol/L) were collected at patients’ homes by fingertip puncture. Samples for phenylalanine were collected on day 2 and day 30 via dried blood spot, whereas concentrations of holotranscobalamin were collected into 0.5 mL K3 EDTA-treated microvettes (MiniCollect^®^ Greiner bio-one, 4550 Kremsmünster, Austria) and in 0.8 mL Serum microvettes (MiniCollect^®^ Greiner bio-one, 4550 Kremsmünster, Austria) for zinc and 25-hydroxyvitamin D on day 3 and day 31. Sampling during baseline and at the supplementation endpoint was staggered in an attempt to reduce patient burden. For each patient, blood specimens were collected at a consistent time of day, usually in a fasted state.

Once collected, samples were sent to accredited laboratories for analysis (Genova Diagnostics Europe, CPA number 3054; Medicheck UK, London, UK. CPA number 2857). Phenylalanine was quantified via high performance liquid chromatography (2695 HPLC Separations Module, Waters). The intra-assay coefficient of variation reported by the manufacturer for phenylalanine was <8% and <10% for tyrosine. Holotranscobalamin and 25-hydroxyvitamin D were determined by electrochemiluminescence, whereas zinc was determined by atomic absorption spectroscopy. The intra-assay coefficient of variation reported by the manufacturer for holotranscobalamin, zinc and 25-hydroxyvitamin D was <2.0%, <1.5% and <3.0%, respectively.

### 2.5. Subjective Mood

Subjective measures of mood were assessed using a “Profile of Mood States” (POMS) questionnaire adapted and validated specifically for the assessment of key mood domains in adults with PKU: Anxiety, Depression, Anger, Activity, Tiredness, and Confusion [24]. The questionnaire consisted of 20 items related to: anxiety (4 items); depression (4 items); anger (3 items); activity (3 items); and tiredness (3 items) where responses are indicated on a 5-point Likert scale (0 (not at all), 1 (a little), 2 (moderately), 3 (quite a bit), 4 (extremely)). From this, domain-specific and combined POMS scores were calculated (combined POMS score range; −12 to 58) whereby higher scores reflect a greater intensity of mood symptoms. Lower scores therefore reflect a reduced intensity of mood symptoms and show an improved mood state. A 3-item confusion subscale, which ranges from 0 to 11, was also included. The questionnaire was completed at baseline (day 0), on day 17 and at supplementation endpoint (day 31).

### 2.6. Compliance

To quantify compliance, patients were asked to record how much of the protein substitute was taken daily compared to that recommended.

### 2.7. Anthropometry

Weight and height were measured during baseline (day 1) and at the end of the intervention period (day 31). Weight was determined to the nearest 0.1 kg, using portable scales. Height was measured to the nearest 0.1 cm using a portable stadiometer. From these parameters, a measure of body mass index (BMI;kg/m^2^) was computed.

### 2.8. Statistical Analysis

All statistical procedures were performed using software package IBM SPSS Statistics v24 IBM SPSS v23.0, Armonk, NY, USA). Data were checked for normal distribution with the use of the Kolmogorov–Smirnov normality test and were log-transformed if appropriate before statistical analysis. Statistical analysis comprised paired samples t-test analysis to determine differences for eating behaviours, nutrient intake and nutritional biomarkers, with values expressed as mean difference ± SEM relative to baseline observations. One-way repeated measures ANOVA was used to assess differences in mood (domain-specific and combined POMS scores) over the study period. Statistical significance was accepted at an α level of *p* < 0.05. All data are presented as mean ± SEM unless otherwise stated.

## 3. Results

In total, 12 patients identified as noncompliant met the inclusion criteria and participated in this study (baseline characteristics displayed in Table 2).

Of these, 9 patients presented with classical PKU and the remaining 3 presented with a milder variant. Two were following a relaxed PKU diet whereas 10 patients followed an unrestricted diet. At baseline, two patients were taking a maximum of 1 protein substitute (20 g protein equivalent) daily, whereas the remaining 10 patients were taking no protein substitutes. Except for one specialist metabolic centre (<600 µmol/L), target blood phenylalanine was <700 µmol/L for all patients. In this study, 91.6% of patients’ (*n* = 11 of 12) historical blood phenylalanine was outside of target range and corroborates previous figures [10]. At the time of recruitment, patients had been noncompliant for an average of 4.5 years (range 1–11 years) and consented to participate for a host of reasons including 1) to try something new (*n* = 3); 2) help return to the PKU diet (*n* = 8); 3) improve nutritional intake (*n* = 4); 4) looking to begin a preconception diet (*n* = 1); 5) improve compliance with protein substitutes (*n* =1); 6) help neurocognitive symptoms (*n* = 2) and 7) contribute to research (*n* = 1).

Owing to difficulties associated with acceptability (taste; *n* = 1) and gastrointestinal tolerance (nausea; *n* = 2), three patients dropped out within the first two weeks of the study. Nine patients therefore completed the study and were subsequently included in the final analysis.

### 3.1. Eating Behaviour and Nutrient Intake

Total protein intake was stable from baseline to the intervention endpoint (66.4 ± 10.4 vs. 70.6 ± 11.0 g/d, respectively; *p* = 0.290). The contribution of natural protein to total protein intake, however, decreased significantly between baseline and the intervention endpoint (66.4 ± 10.4 vs. 55.1 ± 10.4 g/d, respectively; *p* = 0.043). Similarly, protein from protein substitutes increased significantly between baseline and the intervention endpoint (2.2 ± 2.2 vs. 15.6 ± 3.0 g/d, respectively; *p* = 0.004). Because of the above, estimated phenylalanine intake decreased significantly between baseline and the intervention endpoint (3318.5 ± 518.4 vs. 2754.9 ± 519.8 mg/d, *p* = 0.043). Fat [and saturated fat] intake also decreased significantly between baseline and the intervention endpoint (67.1 ± 4.9 [24.2 ± 2.1] vs. 51.5 ± 6.5 g/d [18.1 ± 3.1 g/d], respectively; *p* = 0.019 [*p* = 0.041]). Energy (1611.6 ± 158.3 vs. 1503.2 ± 156.2 kcal/d, respectively; *p* = 0.283) and carbohydrate intake (181.3 ± 21.4 vs. 173.9 ± 18.2 g/d, respectively; *p* = 0.765) remained stable between baseline and at the intervention endpoint.

**Figure 1:**
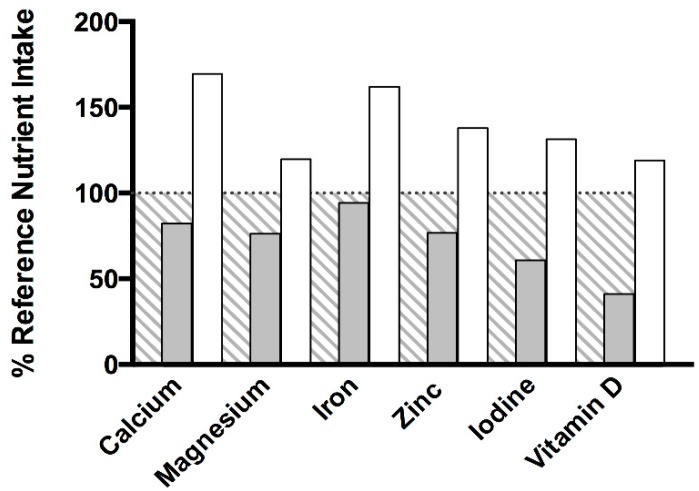
Mean intake of calcium, magnesium, iron, zinc, iodine and vitamin D presented as a percentage compared against the UK reference nutrient intakes to determine nutritional adequacy. Intakes were determined utilising 3-day weighed food records. Grey shaded bars represent intakes during baseline (days 1–3) and white shaded bars represent intakes during the final 3 days of the intervention (endpoint, days 29–31).

At baseline, intakes of calcium (576 mg/d), magnesium (229 mg/d), iron (8.2 mg/d), zinc (7.3 mg/d), iodine (85 µg/d) and vitamin D (4.1 µg/d) were below UK RNI recommendations (Figure 1). For calcium and iron, this was true for 78% (*n* = 7 of 9) of patients. For magnesium and iodine, this was true for 67% (*n* = 6 of 9) and for zinc and vitamin D 89% (*n* = 8 of 9). With daily consumption of the experimental protein substitute, intakes of calcium (1187 mg/d), magnesium (359 mg/d), iron (14.1 mg/d), zinc (13.1 mg/d), iodine (184 µg/d) and vitamin D (11.9 µg/d) at the intervention endpoint increased significantly (*p* ≤ 0.05 for all) and subsequently met UK RNI recommendations (Figure 1). Intakes of magnesium, zinc, iodine and vitamin D were within UK RNI recommendations (RNI) for 78% (*n* = 7 of 9) of patients and 89% for calcium and iron (*n* = 8 of 9). Intakes of thiamin, riboflavin and vitamin C met UK RNI recommendations at baseline, yet intakes increased significantly at the intervention endpoint (*p* ≤ 0.05 for all) with daily consumption of the experimental protein substitute.

### 3.2. Blood Phenylalanine, Tyrosine, Vitamin B12, Zinc and Vitamin D

**Figure 2:**
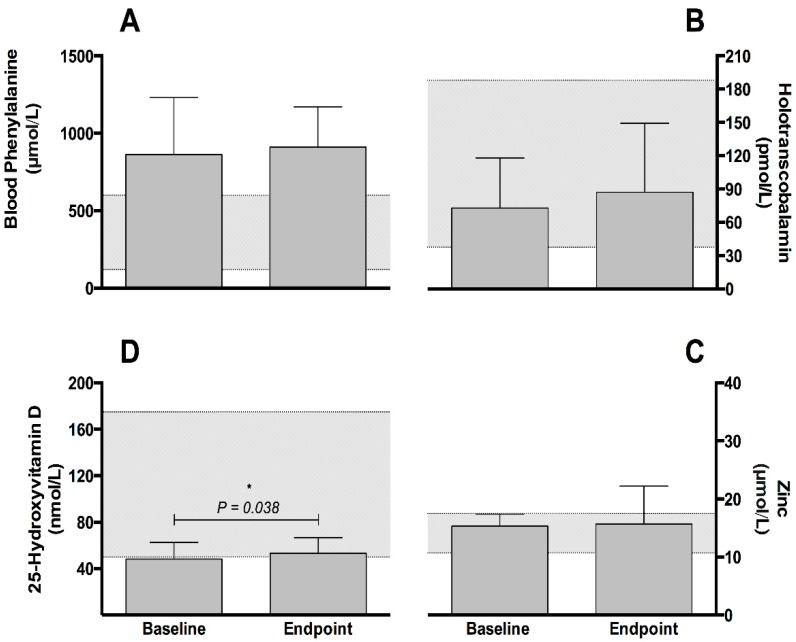
Mean (±SEM) concentrations of blood phenylalanine (µmol/L; panel A), vitamin B12 (holotranscobalamin) (pmol/L; panel B), zinc (nmol/L; panel C) and vitamin D (25-hydroxyvitamin D) (nmol/L; panel D) at baseline and after the intervention period (*n* = 9). For panel A, the shaded area represents the recommended range for blood phenylalanine in PKU [5], whereas the shaded areas for panels B, C and D represent the normal biochemical range for vitamin B_12_ (holotranscobalamin; laboratory (Medicheck UK, London, UK. CPA number 2857) determined range), zinc [25] and vitamin D (25-hydroxyvitamin D) [26].

Blood phenylalanine (882.1 ± 118.3 vs. 893.4 ± 93.3 µmol/L, respectively; *p* = 0.583, Figure 2, panel A) and tyrosine (48.1 ± 11.2 vs. 51.4 ± 16.0 µmol/L, respectively; *p* = 0.594) concentrations remained stable between baseline and at the intervention endpoint.

Concentrations of active vitamin B_12_ (holotranscobalamin) increased 19.8% from baseline (72.6 ± 15.0 pmol/L) to the intervention endpoint (87.0 ± 20.7 pmol/L) but were not significant (*p* = 0.322, Figure 2, panel B). No differences were observed for zinc between baseline and the intervention endpoint (15.3 ± 0.7 vs. 15.7 ± 2.2 µmol/L, respectively; *p* = 0.780, Figure 2, panel C). At baseline, mean vitamin D (25-hydroxyvitamin D) concentrations were below the normal range (50.0 to 175.0 nmol/L). Concentrations of 25-hydroxyvitamin D increased significantly (*p* = 0.038) from baseline (48.3 ± 4.8 nmol/L) to the intervention endpoint (53.3 ± 4.4 nmol/L) and moved within normal range, albeit at the lower end (Figure 2, panel D).

### 3.3. Subjective Mood

Combined POMS score did not differ at any point throughout the study (*p* ≥ 0.05, *n* = 8). For domain-specific analysis, repeated measures ANOVA detected a significant difference over time for feelings of anxiety and confusion (*p* ≤ 0.05). After adjustment for multiple comparisons, reported ratings (and thus intensity of symptom) of anxiety approached significance at day 17 (1.0 ± 0.4, *p* = 0.051) and were significantly lower at the intervention endpoint (1.0 ± 0.3, *p* = 0.048) as compared to baseline (3.3 ± 1.0). For confusion, reported ratings (and thus intensity) of confusion at baseline (2.8 ± 0.9) were again significantly less at day 17 (1.3 ± 0.5, *p* = 0.048), but not at the intervention endpoint (1.5 ± 0.5, *p* = 0.239). See Supplementary Table S1 for full POMS results.

### 3.4. Compliance and Anthropometry

Compliance to the intervention (33 g of the experimental protein substitute in 100 mL of water taken once daily for 28 days) was high at 92.0% ± 6.3%. Body weight (*p* = 0.551) and BMI (*p* = 0.207) remained stable between baseline and the intervention endpoint.

## 4. Discussion

Returning to the PKU diet with strict compliance is extremely difficult. This is particularly true following a period of noncompliance and many patients fail to do this successfully. Strategies aimed at improving PKU diet compliance are therefore needed and may help prevent the metabolic, nutritional and cognitive abnormalities associated with noncompliance, but are greatly lacking. The results presented here demonstrate that reintroduction of a low-volume, nutrient-enriched protein substitute improves eating behaviour and nutrient intake in noncompliant PKU patients. Perceptions of anxiety and confusion also improved yet should be handled as preliminary data. These results have important clinical relevance for metabolic practitioners and may provide a valuable first line approach in patients’ return to the PKU diet. The methodological approach utilised in this study could be used as part of a stepwise approach to optimise the nutritional supply of noncompliant PKU patients with the potential of improving metabolic control through greater compliance over time before further increasing protein substitute servings and restrictions in natural protein intake. While the success of this approach is speculative, the results of this study certainly should be used to further develop a sound stepwise approach to better facilitate a return to the PKU diet.

Management of PKU centres on a lifelong meticulous low protein diet virtually devoid of phenylalanine, where daily phenylalanine-tolerance is usually not greater than 250 to 500 mg (approx. 5 g to 10 g of natural protein) [27]. In the patient population studied, consumption of natural protein and thus dietary phenylalanine at baseline was high (66.4 g/d and 3318.5 mg/d, respectively) and reflective of a metabolically healthy population [28]. Although total protein intakes remained unchanged after introduction of the experimental protein substitute, the contribution of natural protein decreased with corresponding increases in protein from protein substitutes. While intake was still high at the intervention endpoint, natural protein and dietary phenylalanine intake decreased by 11.3 g/d and 565 mg/d and may explain the reductions observed in fat and saturated fat intakes. The introduction of the experimental protein substitute may not have been solely responsible for this, but patients reported having not consciously made any change to their eating behaviours. Intakes of calcium, magnesium, iron, zinc, iodine, vitamin D, thiamin, riboflavin and vitamin C were increased with the experimental protein substitute, as was blood 25-hydroxyvitamin D (by 10.4%) and vitamin B_12_ (by 19.8%). This represents an important outcome as the intake of calcium, magnesium, iron, zinc, iodine, vitamin D at baseline were below the UK RNI recommendations [23]. Evidence of blood- and diet-reported micronutrient insufficiencies supports previous studies in adult PKU patients following a relaxed diet together with relaxed and/or discontinued protein substitute intakes [15,16]. This study also provides evidence that an inadequate intake of these nutrients is evident in patients following an unrestricted diet which has not previously been described, despite reporting higher intakes of natural protein.

While noncompliance in adult PKU patients is widespread [9], the metabolic damage of this may not be immediately noticeable. Noncompliance to the degree as observed at baseline likely bolsters circulating phenylalanine and consequently uptake in the brain [29]. From a physiological perspective, increased phenylalanine uptake in the brain contributes to prefrontal cortex dysfunction [30] and inhibits the production of several neurotransmitters, catecholamines and hormones including dopamine, norepinephrine and serotonin which are involved in regulating mood, emotion, and cognition [31,32]. It is therefore common for noncompliant PKU patients to report increased feelings of inhibition, anxiety, depression and low self-esteem [19,33]. Indeed, recent research imitating noncompliance has provided evidence that high phenylalanine levels directly affect mood [34]. The cited study, however, used a generic POMS questionnaire to quantify mood. A validated and adapted POMS questionnaire for use with PKU adults has since been developed which is both simple to use and elicits minimal patient burden [24] and was used in the present study. Though no differences were detected for blood phenylalanine and combined mood, a reduction in intensity ratings of anxiety and confusion was observed over the course of this study. While this opposes the findings of ten Hoedt et al. [34], it is not inconceivable that the reduction in natural protein intake contributed to the improvements in mood by reducing phenylalanine uptake in the brain and increasing dopamine, norepinephrine and serotonin production, yet this theory remains to be confirmed. Clearly, this was not reflected at a blood level but may have been unsurprising considering only one serving was consumed daily in addition to the short observation period. Mood, and thus neurocognitive function, is considered one of the most important motivating factors determining PKU diet compliance [35] and therefore remains an important area for further study. Compliance with the experimental protein substitute was excellent (92% of the prescribed amount). While no correlations were detected, the excellent compliance may be attributed to the improvements in mood but is most likely due to the one-a-day, low volume presentation and flavour (citrus) of the experimental protein substitute.

These findings are not without limitation. Firstly, caution should be taken when extrapolating the results of this study, as the findings are constrained to a relatively small number of noncompliant PKU patients over a short observational period yet remain comparable to other studies in rare metabolic diseases, especially studies concerning noncompliant patients [13,14]. Although findings may be a consequence of bias and/or the placebo effect, a follow-up study of longer duration (e.g., >6 months) with an increased number of noncompliant patients should be performed before concrete conclusions can be drawn. Secondly, food intake, compliance and mood data were obtained through self-report, and blood sampling was obtained through self-completed blood samples. While the authors believe that this closely reflects typical free-living care, patient-centred data collection presents opportunities for bias and misreporting, or indeed could be a product of the placebo effect. The ability to conduct robust scientific research in the field of rare inherited metabolic disorders, however, is particularly difficult, and while the prevalence of noncompliance in PKU is widespread, many noncompliant patients are disengaged from active metabolic follow-up, let alone taking part in relatively complicated research procedures. This made recruitment particularly difficult. This study was initially designed as a randomised control trial (with 2 arms; experimental protein substitute vs. habitual noncompliant practice (control arm)), yet due to issues experienced with recruitment we were unable to collect data of a satisfactory number to the control arm (*n* = 2) to be included in analysis and therefore only reported data from the intervention arm. Patients in this study may therefore only represent a sub-set of noncompliant PKU patients who are motivated to either reinstate strict dietary compliance or contribute to research.

## 5. Conclusions

This study begins to suggest that the introduction of a low-volume, nutrient-enriched protein substitute can be effectively used to re-engage noncompliant PKU patients in their dietary management through a low burden regimen with immediate nutritional and possible mood benefits. The data may begin to support the use of a stepwise approach to aid the return to the PKU diet, with the long-term clinical aim of achieving strict compliance with blood phenylalanine levels in recommended ranges and intake of a nutritionally complete diet, yet further work is needed to confirm this. These results are very encouraging and should stimulate metabolic practitioners to engage with their noncompliant patients to encourage them to consider returning to the PKU diet. They should also prompt future research concerned with developing evidence-based strategies to make the return to the PKU diet easier for patients, with the approach described here as a foundation. Considering the nutritional and health-related implications of noncompliance, frequent monitoring of nutritional intake and mood should be incorporated as part of metabolic follow-up in a programme that continues for life.

## Figures and Tables

**Table 1:** Nutritional composition of the experimental protein substitute.

**Per 33 g Serving**		
Energy value	kJ (kcal)	414 (98)
Protein equivalent	g	20.0
Carbohydrate	g	3.5
Fat	g	0.3
Docosahexaenoic acid	mg	100
**Vitamins**		
Vitamin A	µg RE	730
Vitamin D_3_	µg	14.0
Vitamin E	mg-α-TE	7.0
Vitamin K	µg	35.0
Thiamin	mg	0.80
Riboflavin	mg	1.0
Niacin	mg	7.6
Pantothenic acid	mg	3.0
Vitamin B_6_	mg	0.83
Folic acid	µg	124
Vitamin B_12_	µg	2.2
Biotin	µg	23
Vitamin C	mg	70
**Minerals**		
Sodium	mg	9.9
Potassium	mg	200
Calcium	mg	840
Magnesium	mg	200
Iron	mg	10
Zinc	mg	10
Manganese	mg	1.8
Molybdenum	µg	35
Selenium	µg	25
Chromium	µg	32
Iodine	µg	124

**Table 2:** Patient characteristics ^1^.

Characteristics	Means ± SEMs
Age, year	33.7 ± 2.6
Weight, kg	84.6 ± 6.7
Height, cm	163.5 ± 1.9
BMI, kg/m^2^	31.6 ± 2.4
Duration of noncompliance, year	4.5 ± 1.0
**Blood phenylalanine**	
Historical phenylalanine µmol/L ^2^	1059.7 ± 101.8
Baseline phenylalanine µmol/L ^3^	882.1 ± 118.3

^1^ Patient characteristics are presented for *n* = 12. ^2^ Mean blood phenylalanine concentration from past 3 historical blood phenylalanine tests (*n* = 11) prior to recruitment. ^3^ Data are presented for patients who completed the study and included in the final analysis (*n* = 9). BMI: body mass index.

## Supplementary Materials

The following are available online at https://www.mdpi.com/2072-6643/11/9/2035/s1, Table S1: Subjective measures of mood.
