# Supplementary material for: Improved Eating Behaviour and Nutrient Intake in Noncompliant Patients with Phenylketonuria after Reintroducing a Protein Substitute: Observations from a Multicentre Study
Source: Nutrients. 2019 Aug 30;11(9):2035. doi: 10.3390/nu11092035 (PMC6770397; doi:10.3390/nu11092035)
Supplement: Supplementary file 1 [file nutrients-11-02035-s001.pdf]

Supplementary table S1

Improved Eating Behaviour and Nutrient Intake in Noncompliant Patients with Phenylketonuria after Reintroducing a Protein Substitute; Observations from a Multicentre Study

Supplementary Table. Subjective measures of mood.

Mood state	Day 1	Day 17	Day 31	P value[1]	P value[2]	P value[3]
Activity	4.2±1.0	4.8±1.4	5.0±1.0	P = 0.620	P = 0.451	P = 0.741
Anger	1.1±0.6	0.9±0.4	1.6±1.1	P = 0668	P = 0.708	P = 0.365
Anxiety	3.3±1.0	1.0±0.4	1.0±0.3	P = 0.050	P = 0.048*	P = 1.000
Confusion	2.8±0.9	1.3±0.5	1.5±0.5	P = 0.048*	P = 0.265	P = 0.711
Depression	2.0±1.6	0.9±0.5	2.1±1.3	P = 0.357	P = 0.956	P = 0.393
Tiredness	3.9±1.6	2.1±0.6	1.8±1.1	P = 0.314	P = 0.342	P = 0.662
Combined mood	6.6±4.4	0.5±2.1	0.9±3.3	P = 0.175	P = 0.357	P = 0.880

[1]Baseline compared to day 17. [2] Baseline compared to day 31. [3] Day 17 compared to day 31. * signifies a significant difference of $P<0.05$.
